# Partial penectomy for severe penile ulceration caused by cholesterol crystal embolization

**DOI:** 10.1002/iju5.12631

**Published:** 2023-08-25

**Authors:** Toshiki Matsushima, Kensuke Mitsunari, Nobuhide Maekawa, Shota Yamada, Hidenori Ito, Hiroki Kurata, Yuichiro Nakamura, Tomohiro Matsuo, Kojiro Ohba, Ryoichi Imamura

**Affiliations:** ^1^ Department of Urology Nagasaki University Graduate School of Biomedical Sciences Nagasaki Japan

**Keywords:** cholesterol crystal embolism, partial penectomy, penile ulceration

## Abstract

**Introduction:**

Cholesterol crystal embolism is a rare microembolic disease caused by cholesterol crystals that can present with various symptoms after vascular surgery, catheterization, or anticoagulation therapy. We report a case of penile ulceration caused by cholesterol crystal embolism.

**Case presentation:**

A 72‐year‐old man undergoing maintenance dialysis for end‐stage renal failure presented with penile pain and a black glans ulcer. Despite low‐density lipoprotein apheresis, he was referred to our hospital because of lack of improvement. Based on his medical history and clinical presentation, including artificial vascular replacement and right toe amputation, cholesterol crystal embolism was suspected and partial penectomy was performed, thus confirming the diagnosis. Penile pain resolved after surgery, and he was discharged on Day 10. Unfortunately, he died after small bowel perforation developed 2 months after surgery.

**Conclusion:**

Penile ulcers caused by cholesterol crystal embolism may indicate the severity and progression of disease and typically require surgical intervention.

**Keynote message**

Cholesterol crystal embolism‐induced penile ulcers require early consideration as the differential diagnosis and often require surgical intervention. Conservative treatment is usually ineffective. It is important to improve outcomes through case accumulation. These ulcers may reflect the disease severity and prognosis.

**Abbreviations & Acronyms**
AA: aortic aneurysm
AAA: abdominal aortic aneurysm
AF: atrial fibrillation
AS: aortic stenosis
ASO: arteriosclerosis obliterans
CAG: coronary angiography
CCE: cholesterol crystal embolism
DL: dyslipidemia
DM: diabetes mellitus
EVAR: endovascular aortic repair
HD: hemodialysis
HT: hypertension
LDL: low‐density lipoprotein
MR: mitral regurgitation
PF: precipitating factor

## Introduction

CCE is a systemic microembolic disease triggered by cholesterol crystals that can present with various symptoms and is often associated with major vascular surgery, intravascular catheterization, or anticoagulation therapy.^1^ Although skin, renal, and gastrointestinal symptoms are commonly reported, penile ulceration caused by CCE is rare. This report describes a case of CCE‐induced penile ulceration.

## Case presentation

A 72‐year‐old man with a history of chronic renal failure who was undergoing maintenance dialysis for end‐stage renal failure presented with penile pain and a black ulcer on the glans penis. His medical history included chronic renal failure, acute myocardial infarction, AAA, bilateral toe gangrene, small bowel perforation, HT, and DL. He had previously undergone percutaneous coronary intervention for acute myocardial infarction, artificial vascular replacement surgery for left iliac artery stenosis, and right toe amputation because of CCE. Subsequent repeated gangrene development in his toes required multiple amputations. Dual antiplatelet therapy was required for 6 months after acute myocardial infarction. Thereafter, single‐agent antiplatelet therapy was required. Despite LDL apheresis for penile pain, he was referred to our hospital because of lack of improvement.

**Fig. 1:**
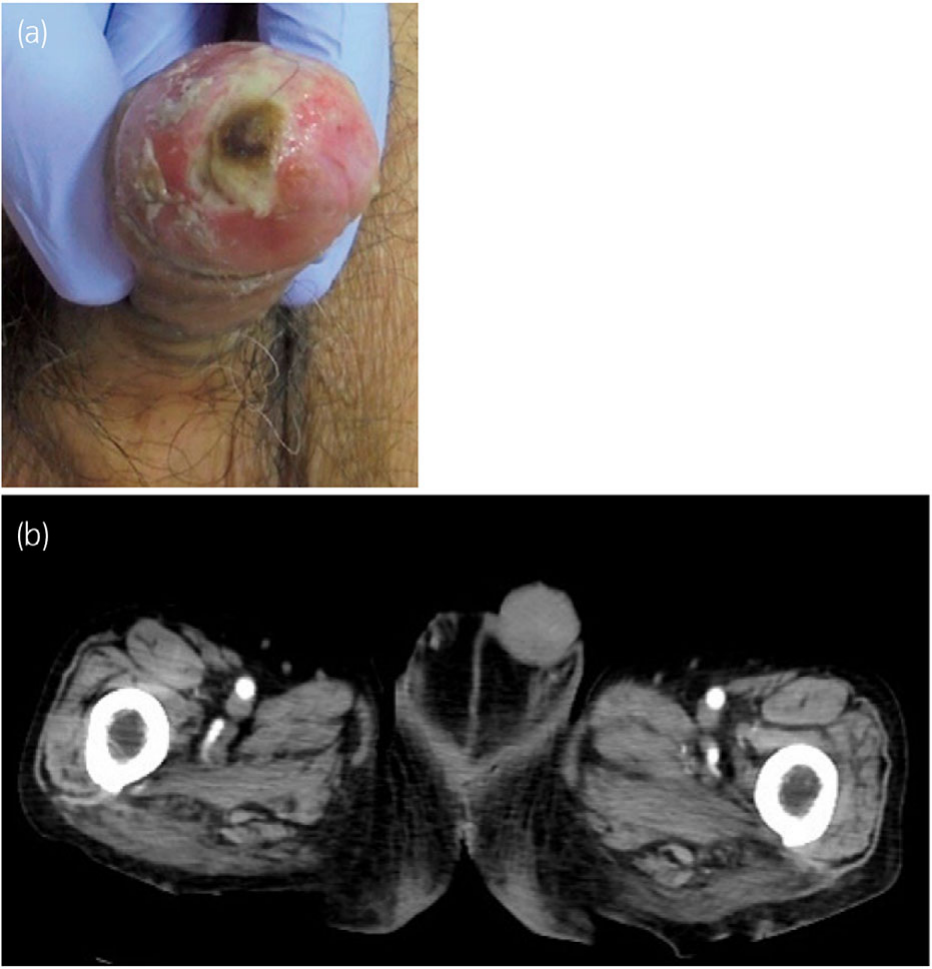
(a) A 10‐mm black ulcer is observed on the glans penis. (b) Contrast‐enhanced computed tomography image shows no evidence of penile ischemia.

A black ulcer measuring 10 mm on the glans penis was observed during the physical examination (Fig. 1a). Laboratory findings revealed a white blood cell count of 8800/μL, C‐reactive protein level of 0.91 mg/dL, and no signs of inflammation. The serum total cholesterol level was 105 mg/dL despite LDL apheresis. Testing to determine sexually transmitted infections yielded negative results. Contrast‐enhanced computed tomography showed no significant differences between the black ulcerated area of the glans penis and the rest of the penis; therefore, we concluded that the likelihood of penile ischemia was low (Fig. 1b).

Although malignancy was not ruled out completely as the differential diagnosis, CCE and other conditions that cause penile ulcers, such as calciphylaxis, were strongly suspected based on his medical history and clinical findings. However, because of his severe penile pain, conservative treatment was not an option. Therefore, we explained the risks and benefits to patient and decided to perform a partial penectomy. We did not perform a histopathological examination to determine the tissue diagnosis.

**Fig. 2:**
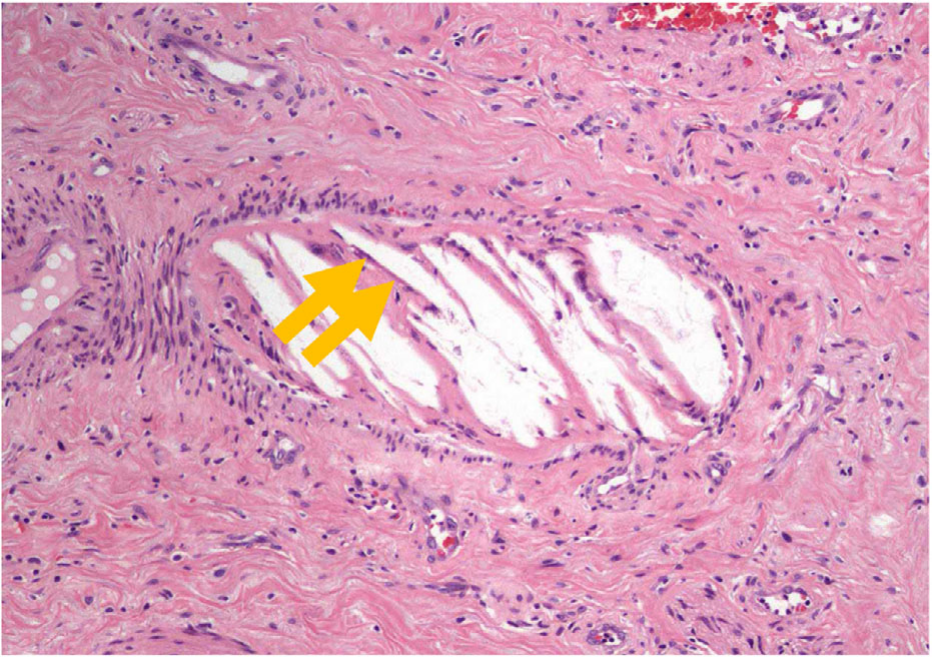
Photomicrograph showing histological findings (hematoxylin and eosin staining).

During partial penectomy, an adequate margin of approximately 3 cm around the ulcer was ensured to allow for the preservation of approximately 50% of the penile tissue. A pathological examination revealed epidermal detachment, necrotic tissue adherence, and inflammatory exudate in the ulcer. Moreover, cholesterol clefts with thrombotic occlusions were observed in the surrounding vessels, and the diagnosis of CCE was confirmed (Fig. 2).

After surgery, the penile pain gradually decreased and eventually disappeared. Despite the presence of hypoalbuminemia, the postoperative course was relatively favorable. The patient was discharged on Day 10. However, he died after the development of small bowel perforation 2 months after surgery.

## Discussion

CCE is mainly caused by the rupture of atheromatous plaques in large arteries, such as the abdominal aorta, iliac artery, and femoral artery, which leads to the occlusion of small peripheral arteries by cholesterol crystals, resulting in thromboembolism in multiple organs.^2^, ^3^ Natural cases are rare. Approximately 80% of cases are induced by factors such as vascular catheterization, major vascular surgery, and anticoagulant therapy with warfarin, heparin, urokinase, and thrombolytic therapy. CCE occurs more frequently in elderly men with risk factors such as HT, atherosclerotic cardiovascular disease, renal failure, and AA.^4^ Symptoms include renal dysfunction (50%) and skin symptoms such as livedo reticularis, which is a net‐like rash that occurs on the lower limbs, ulcers, and gangrene (34%).^1^, ^4^ Treatment for CCE has not yet been established; therefore, symptomatic treatment to improve microcirculation and reduce pain, including stopping the triggering examination or anticoagulant therapy, is mainly used.^5^ Renal and skin symptom improvement have been observed with steroid therapy,^6^, ^7^ and the effectiveness of statins, which have plaque‐stabilizing and anti‐inflammatory effects,^8^ and LDL apheresis^9^ have been reported; however, these improvements have been described only by case reports. LDL apheresis was performed to treat this case of CCE, but the penile ulceration symptoms and findings caused by CCE did not improve. The appearance of penile ulceration associated with CCE may indicate more severe disease progression, as demonstrated by the lack of improvement after LDL apheresis.

CCE‐induced penile ulcers are rare; including the present case, only 10 cases have been reported (Table 1).^10^, ^11^, ^12^, ^13^, ^14^, ^15^, ^16^ The median age of patients with such cases is 67.5 years (range, 56–82 years), and most cases involve HT and DL. Renal impairment was observed in seven of the 10 reported cases, and skin lesions (particularly on the feet and toes) were present in all cases. For eight of the 10 cases, onset occurred after angiography or intravascular surgery; however, the remaining cases developed without an apparent cause. All but two cases underwent surgical excision to treat the penile ulcers, which were histologically confirmed to be caused by CCE. No clear postoperative complications were observed; however, seven patients died within 6 months after surgery because of infections or gastrointestinal perforation. Although histological evidence was not obtained, based on the reports of bowel perforation caused by CCE, it is plausible to consider CCE as the cause of bowel perforation and subsequent death. This suggests that the appearance of penile ulceration caused by CCE may be a poor prognostic factor, even with surgical treatment. However, it is considered important for pain management purposes.

**Table 1:** Summary of 10 reported cases of CCE‐induced penile ulcers

Author	Year	Age, years	Underlying disease	PF	Renal failure	HD	Skin lesions	Interval between skin lesion onset and penile necrosis	Diagnosis	Surgery	Outcome
Rosansky^10^	1982	66	ASO	Bypass	Yes	—	Yes	0	Clinical diagnosis	No	Dead
		56	HT, MR. DL	Angiography	Yes	—	Yes	2–3 days	Pathology specimen	Yes	Dead
		58	ASO, DM	Angiography	No	—	Yes	5 days	Pathological anatomy	Yes	Dead
Scholten^11^	1995	82	None	None	No	No	Yes	2–3 weeks	Pathology specimen	Yes	Alive
Takats^12^	1995	73	AF, AS, HT	CAG	Yes	Yes	Yes	0	Clinical diagnosis	No	Dead
Quintart^13^	1997	69	AA	Angiography	Yes	No	Yes	2–3 days	Pathology specimen	Yes	Dead
Mondragon^14^	1998	65	DL, DM, HT	None	Yes	Yes	Yes	6 months	Pathology specimen	Yes	Dead
Bettina^15^	2002	56	AAA, DL, HT	CAG, EVAR	Yes	No	Yes	3 weeks	Pathology specimen	Yes	Alive
Bhasin^16^	2004	71	DL, DM, HT, Angina	CAG	No	No	Yes	0	Pathology specimen	Yes	Alive
Current case	2022	72	AAA, DL, HT	CAG, EVAR	Yes	Yes	Yes	6 years	Pathology specimen	Yes	Dead

CCE‐induced penile ulcers are believed to result from thrombosis in the peripheral artery of the penis, which branches from the internal pudendal artery.^16^ In this case and seven cases reported previously,^10^, ^11^, ^13^, ^14^, ^15^ foot ulcers had preceded the development of penile ulcers, indicating that foot ulcers often precede penile ulcers in cases of CCE. The penis is rarely affected by ischemia because of its abundant collateral circulation,^3^ and it is thought that penile ulcers occur after the progression of CCE after foot ulceration. Therefore, a history of foot ulcers may be useful to the differential diagnosis of penile ulcers. CCE‐induced penile ulcers may reflect the severity and progression of disease. Conservative treatment is often ineffective; therefore, surgical intervention is required. Early consideration of this disease for the differential diagnosis is important. CCE‐induced penile ulcers indicate a poor prognosis; therefore, it is important to improve treatment outcomes through the accumulation of cases.

## Conclusion

CCE‐induced penile ulcers may reflect the severity and progression of disease and often require surgical intervention.

## Author contributions

Toshiki Matsushima: Writing – original draft. Kensuke Mitsunari: Project administration; supervision; writing – original draft; writing – review and editing. Nobuhide Maekawa: Writing – review and editing. Shota Yamada: Writing – review and editing. Hidenori Ito: Writing – review and editing. Hiroki Kurata: Writing – review and editing. Yuichiro Nakamura: Writing – review and editing. Tomohiro Matsuo: Writing – review and editing. Kojiro Ohba: Writing – review and editing. Ryoichi Imamura: Supervision; writing – review and editing.

## Conflict of interest

The authors declare no conflict of interest.

## Approval of the research protocol by an Institutional Reviewer Board

No ethics approval was required for this case report.

## Informed consent

Written informed consent was obtained from the patient.

## Registry and the Registration No. of the study/trial

Not applicable.
